# Incidence of Brain Vascular Damage in a Population With Parkinson's Disease: Statistical Comparison by Age Subassemblies With Age Homogeneous Control Groups

**DOI:** 10.7759/cureus.8778

**Published:** 2020-06-23

**Authors:** Flora Zarola

**Affiliations:** 1 Neurology, Unit of Parkinson's Disease and Movement Disorders, Azienda Sanitaria Locale (ASL), Albano Laziale, ITA

**Keywords:** essential tremor, vascular parkinsonism, stroke, brain vascular disease, aging, neuroimaging, parkinson's disease, juvenile parkinson's

## Abstract

We recently published studies on the incidence significance of cerebrovascular disease (CVD) in Parkinson's disease (PD) and some related movement disorders. The CVD was assessed by including lesions morphologically diagnosed by magnetic resonance imaging (MRI) or computed tomography (CT) scan studies that defined ischemic vascular damage with a range from leukoaraiosis to multiple microinfarcts with various distribution, minor stroke, and major stroke and hemorrhagic lesions. These lesions were detected at the beginning of Parkinsonian symptoms or earlier; in any case, all the patients were diagnosed as PD patients according to the international classification and response to therapy. These studies have shown in a large age-range (40-96 years old) group of patients with PD that CVD was more severe and extensive if compared to a control population that is relatively homogeneous for age. Furthermore, carrying out the same comparison with other extrapyramidal diseases, such as essential tremor (ET), this statistical significance was still present while it was not when the investigation was performed between ET and the same control group. Therefore, we decided to deepen the study about the incidence of CVD in the different groups by adding a higher level, namely, considering different age subassemblies. We carried out this study because it is commonly assumed that CVD is a simple comorbidity in aged patients, so it was interesting to check if this pathology was present even in a younger age group.

## Introduction

Recent studies about the statistical significance of the incidence of cerebrovascular disease (CVD) in Parkinson's disease (PD) pointed out the fact that CVD was not a simple comorbidity when compared to some related movement disorders or age homogeneous control populations [1-5]. CVD was assessed by including lesions morphologically diagnosed by magnetic resonance imaging (MRI) or computed tomography (CT) scan studies, which defined ischemic vascular damage with a range from leukoaraiosis to multiple microinfarcts with various distributions, minor stroke, major stroke, and hemorrhagic lesions. These lesions were detected at the beginning of Parkinsonian symptoms or earlier; in any case, all the patients were diagnosed as PD patients according to the international classification and response to therapy. In a large age-range group of patients with PD, we demonstrated that CVD was more severe and extensive if compared to a control population fairly homogeneous for age [1-3]. Furthermore, carrying out the same comparison of the PD group with other extrapyramidal diseases, such as essential tremor (ET), the statistical significance of CVD in PD was surprisingly still present, even though, most of the time, the kinetic tremor is defined as 'atherogenic' tremor in senile patients, while it was not when the investigation was performed between ET and the same control group [2]. Therefore, we decided to deepen the investigation about the incidence of CVD in the different groups by adding a higher level, namely, considering different age subassemblies. We carried out this study because it is commonly assumed that CVD is a simple comorbidity in aged patients, so we wanted to check if this pathology was present even in a younger age group or if the significance in the incidence of CVD was due to the statistical weight of older patients spread on the entire population.

## Materials and methods

A total of 72 patients with defined PD was examined. The control population consisted of 117 outpatients with other pathological conditions. All patients were selected because they had also been studied morphologically for various clinical reasons with brain MRI. There were no patients with definite genetic PD. Both groups were then divided for statistical analysis into three bands. The control groups consisted of a younger one of 18 patients, age ranging from 40 to 60 years old (median age 59), an intermediate band of 40 patients ranging from 61 to 74 years old (median age 69), and, finally, an elderly group of 77 patients ranging from 75 to 92 years old (median age 85). The PD group consisted of a younger subassembly of five patients ranging from 54 to 60 years old (median age 59), an intermediate group of 30 patients ranging from 61 to 74 years old (median age 71), and a senior group of 37 patients from 75 to 96 years old (median age 81). Statistical analysis was carried out using chi-square tests for association between two categorical variables cross-comparing each age subgroup affected and not affected by CVD. For the younger group, the comparison chi-square was 1.8315, the p-value was 0.17595 (not significant at p < 0.05), the chi-square with Yates correction was 0.6593, and the p-value was 0.416793 (not significant at p < 0.05); for the intermediate group, the comparison chi-square was 7.0844, the p-value was 0.007776 (significant at p < 0.05), the chi-square with Yates correction was 5.8395, and the p-value was 0.015671 (significant at p < 0.05); for the elderly group comparison, the chi-square was 5.59, the p-value was 0.018063 (significant at p < 0.05), the chi-square with Yates correction was 4.5453, and the p-value was 0.03301 (significant at p < 0.05).

## Results

As can be seen, there are three levels of statistical significance. In Table 1, there is no significance in the incidence of CVD between the control population and the PD group; indeed, notice that the PD population is very small and weak as concerns statistical weight. Despite all this, it is noticeable that three out of five patients show severe CVD comorbidity; these three patients had the diagnosis of PD and CVD four years before the present statistical analysis or at an even younger age. This evidence is in favor of vascular involvement in the younger age group in the possible pathogenesis of PD. Moreover, this lack of significance is congruous with the commonly accepted hypothesis of prevailing multifactorial pathogenesis of PD extraneous or more relevant with respect to CVD as numerous extensive studies in progress for many years (including genetic etiology analysis) claimed. In the other two tables (Table 2 and Table 3), there is instead a strong significance of the incidence of CVD in patients with PD as compared to homogeneous control populations; in the older age group, mostly chronic vascular damage could play a more important role concerning the duration of vascular disease. This result includes patients with a relatively lower elderly age. Note that in the last two groups, the number of subjects is broader and more comparable between controls and PD patients. This analysis confirms that CVD may play an important role in the pathogenesis of PD, as already described in previous studies less detailed as concerns the age of the investigated population [1-4,6-9]. There is a possible bias regarding the comorbidity of CVD in elder age as a simple age-related coincidence, while in this study, even the ‘younger’ patients show neuroimaging signs of vascular brain damage like microvascular or more severe ischemic patterns, as issued in common clinical practice.

**Table 1:** Younger subassembly PD versus controls CVD comparison CVD, cerebrovascular disease; PD, Parkinson’s disease Note: Chi-square is 1.8315. p-value is .17595 (not significant at p < .05). Chi-square with Yates correction is 0.6593. p-value is .416793 (not significant at p < .05).

	CVD yes	CVD no	Marginal row totals
Control 40 - 60 years	4	11	15
PD 54 - 60 years	3	2	5
Marginal column totals	7	13	20

**Table 2:** Intermediate subassembly PD versus controls CVD comparison CVD, cerebrovascular disease; PD, Parkinson’s disease Note: Chi-square is 7.0844. p-value is .007776 (significant at p < .05). Chi-square with Yates correction is 5.8395. p-value is .015671 (significant at p < .05).

	CVD yes	CVD no	Marginal row totals
Control 61 - 74 years	18	22	40
PD 61 - 74 years	23	7	30
Marginal column totals	41	29	70

**Table 3:** Elderly subassembly PD versus controls CVD comparison CVD, cerebrovascular disease; PD, Parkinson’s disease Note: Chi-square is 5.59. p-value is .018063 (significant at p < .05). Chi-square with Yates correction is 4.5453. p-value is .03301 (significant at p < .05).

	CVD yes	CVD no	Marginal row totals
Control 75 - 92 years	53	24	77
PD 75 - 96 years	33	4	37
Marginal column totals	86	28	114

## Discussion

Classic studies on PD pathogenesis have increasingly pointed out the importance of the enlarging amount of neuronal damage at the pre-synaptic level that pre-clinically occurs with the loss of the nigrostriatal system [10-11]. This damage may be preceded by, for many years in a chronic way, the loss of receptors at the level of nigrostriatal neurons until the amount of loss necessary for the clinical expression of the disease is evident. A pathogenetic role of vascular damage can be hypothesized for this process based on the statistical analyses and was observed in past studies on animals [6-9]. Hence, the observational studies supplied with enough clinical data can provide an important attestation of a clinical suspicion frequently occurring in current daily practice, as arose in the present study when the author noticed a considerable vascular lesion load in the younger patients with PD, such as the ones included in this study and cited in materials and methods. In those patients, CVD was already detected at the beginning of therapeutic treatment, as multiple random vascular infarcts that were noted both in deep and hemispheric white and gray matter (i.e., without an immediate morphological link with the nigrostriatal system). However, the patients stabilized and responded to dopaminergic therapy. This finding could lead to interpreting the visible vascular lesions as macroscopic sentinels of chronic ischemic damage, remarkably acting at a biochemical pre-synaptic level in the nigrostriatal network. Note that in the majority of cases, this morphological finding can lead the physician to diagnose the patient as having vascular Parkinsonism, both if younger or aged, with poor therapeutic attempts. Moreover, in our clinical experience, countless patients started suffering from PD after an ischemic or hemorrhagic stroke.

## Conclusions

Current clinical practice and accepted classifications generally agree to separate PD from vascular Parkinsonism sharply. This is evidently in contrast with the common assertion that PD etiology, excluding an overt genetic component, is uncertain. Our present and previous studies lead us to reconsider the current classifications of PD and extrapyramidal diseases to include the relevance of vascular pathogenesis in a spectrum of central nervous system affections responding or not responding to dopaminergic therapy, thus even redefining the nosological entity of PD. By splitting the patient population into various age subassemblies, there were emerging data in favor of the hypothesis prospected: the statistical analysis in a given large population shows an instant photograph of the statistical influence of the considered parameter (CVD) while the prevalence of the pathogenetic cause is more evident in chronic CVD as long as it lasts.
